# Remote Identification of Sheep with Flystrike Using Behavioural Observations

**DOI:** 10.3390/ani9060368

**Published:** 2019-06-18

**Authors:** Emily P. Grant, Sarah L. Wickham, Fiona Anderson, Anne L. Barnes, Patricia A. Fleming, David W. Miller

**Affiliations:** 1 College of Science, Health, Engineering & Education, Murdoch University, Perth, WA 6150, Australia; sarahlwickham@outlook.com (S.L.W.); F.Anderson@murdoch.edu.au (F.A.); A.Barnes@murdoch.edu.au (A.L.B.); T.Fleming@murdoch.edu.au (P.A.F.); D.Miller@murdoch.edu.au (D.W.M.); 2 Cooperative Research Centre for Sheep Industry Innovation (Sheep CRC), Armidale, NSW, 2350, Australia

**Keywords:** flystrike, sheep, behaviour, qualitative behavioural assessment (QBA), on-farm assessment

## Abstract

**Simple Summary:**

Flystrike in sheep is a common condition in Australia where parasitic flies lay eggs on soiled wool or open wounds; and the resulting maggots feed off the flesh. Identification of ‘flystruck’ individuals is crucial for treatment; but requires labour-intensive physical examination of every animal. The aim of this study was to investigate the behaviour of sheep; while they remained in the paddock; to try and visually distinguish those suffering from flystrike. Observers who were blinded to the flystrike status of the sheep were asked to score the animal’s body language from video footage. These scores were then compared with the condition of the wool and whether the sheep were flystruck. The observers found that the flystruck sheep exhibited behavioural characteristics that corresponded to the flystrike severity and the condition of the wool around the tail (breech) of the sheep. We therefore conclude that behavioural monitoring of sheep in the paddock could be used to identify animals that had flystrike.

**Abstract:**

Flystrike is a major problem affecting sheep in Australia. Identification of ‘flystruck’ individuals is crucial for treatment; but requires labour-intensive physical examination. As the industry moves toward more low-input systems; there is a need for remote methods to identify flystruck individuals. The aim of this study was to investigate the behaviour of sheep with breech flystrike within a paddock setting. Video footage of sixteen Merino sheep; eight later confirmed with flystrike and eight without; was collected as they moved freely within the paddock with conspecifics. Quantitative behavioural measurements and a qualitative behavioural assessment (QBA) were conducted and compared to their breech conditions (i.e., faecal/urine staining; flystrike severity). Both qualitative and quantitative assessments indicated behavioural differences between flystruck and non-flystruck animals. Flystruck sheep had a behavioural profile characterised by restless behaviour; abnormal postures and reduced grazing time (*p* < 0.05). Furthermore; flystruck sheep were scored to have a more ‘exhausted/irritated’ demeanour using QBA (*p* < 0.05). The behavioural responses also corresponded to the flystrike severity scores and condition of the breech area. We conclude that remotely assessed behaviour of flystruck sheep diverges markedly from non-flystruck sheep; and thus could be a low-input method for identifying and treating affected animals.

## 1. Introduction

Flystrike, or cutaneous myiasis, is a major health and welfare problem within the Australian sheep industry. The disease of flystrike is caused by the chemical and mechanical effects of blowfly (subfamily Calliphoridae) larvae (maggots) as they feed on the host’s dermal tissue and inflammatory exudates [1,2,3,4]. Onset of disease is rapid [5,6], and is characterised by cutaneous lesions, pyrexia (fever), inflammation and the severe irritation of the skin [4,7]. Infested sheep can experience reductions in feed intake, body weight, wool production and lamb losses [6,8]. More important, however, is the risk of death from bacterial and/or systemic toxaemia in severe or untreated cases [8,9]. It stands to reason that, when not properly managed, flystrike represents a debilitating disease that raises significant welfare concerns.

Presently, to minimise stock susceptibility, sheep producers rely on strategies that incorporate both preventative treatments such as drenching, spraying with chemical treatments, crutching and shearing, and the management of factors that predispose animals to flystrike, such as gastrointestinal parasites and diarrhoea [10,11]. However, such management strategies are often labour-intensive and costly. Moreover, due to recent opposition to mulesing (removing folds of skin from the breech area to reduce risk of flystrike), producers have been encouraged to reduce their dependence on this surgical practice; thus, there is heavy reliance on the use of insecticides to manage flystrike [11]. These management strategies do not appear to offer long-term or reliable protection against severe outbreaks of flystrike [12], however, and frequent physical examination of penned animals is fundamental, particularly during severe flystrike seasons typically seen during the Spring months. Such monitoring imposes significant costs to producers in terms of time and labour. The development of simple, animal-based, within-paddock (remote) indicators would give producers a tool to aid decision making regarding management of flystrike. Currently, no formal protocols are available that target remote visual identification of flystruck sheep.

Behaviour plays an important role in the diagnosis and early detection of health issues and diseases [13,14]. There is some evidence to suggest that flystruck sheep display behaviours indicative of agitation [15]. However, to date there have been no studies that focus on the application of behavioural assessments for the early identification of flystrike in sheep. Animal behaviour is complex and dynamic, and as such it stands to reason that assessments should be comprehensive and endeavour to reflect the state of the whole animal. Qualitative Behavioural Assessment (QBA) has been proposed as one such ‘whole-animal’ measure, with observers providing assessments of expressive behaviour (body language) through the integration and summary of details of behaviour, posture and movement, and context [16,17,18]. The aim of this study was to investigate the behaviour, using QBA and quantitative measures, of sheep with and without breech-strike within a paddock setting. Differences in QBA scores for sheep with flystrike may be useful as an indicator for further management options to improve their health and welfare.

## 2. Materials and Methods

This experiment was approved by the Animal and Human Ethics Committees at Murdoch University (R2598/13; N2779/15; O2780/15; 2008/021) and the Animal Ethics Committee of the Department of Agriculture and Food Western Australia (AEC 1-14-02) to ensure compliance with the guidelines of the Australian Code of Practice for the Care and Use of Animals for Scientific Purposes, the Australian Code for the Responsible Conduct of Research 2007, and the National Statement on Ethical Conduct in Human Research, 2007.

### 2.1. Animals and Experimental Design

The behaviour of 16 mature Merino ewes categorised into two groups based on physical examination as flystruck (*n* = 8) and non-flystruck (*n* = 8), were assessed from video footage collected in the paddock. Video footage was collected from ewes within three paddocks (200–300 individuals) during routine inspections for flystrike over a 7-day period. During the inspections, a dedicated and trained member of the research team was responsible for the remote (~50 m distance) visual identification of suspected flystruck and non-flystruck animals for filming and subsequent physical examination. Sheep positively identified as being flystruck by trained personnel, i.e., by the presence of maggots and/or cutaneous lesions, were treated immediately after filming, with the wool around the flystrike area clipped and treated with a short-acting, kill-on-contact insecticide, Extinosad^®^ (Elanco Animal Health, Australia) as per the manufacturer’s recommendations. Those sheep that were categorised as non-flystruck at visual inspection were not treated. Weather conditions for the duration of this study were consistent and there were no apparent unusual climactic events that could bias behavioural recording. Although multiple types of flystrike were recorded, the behavioural assessment of flystruck sheep in the present study was confined to breech flystrike (seven animals) or breech and rump flystrike (one animal), where breech is defined as the area around the tail.

### 2.2. Breech Soiling Assessment

Indicators of breech soiling: dag, dag moisture and urine stain scores, were collected by trained personnel at the time of physical examination in the present study. Breech soiling with faeces (dags), dag moisture content and urine stain were scored using a five-point scoring method [19,20], in which 1 denoted no dags, dry dags and no stain; and 5 was the highest score for each trait, indicating extensive dags, very wet dags and extensive urine staining, respectively. With regard to urine stain, severity was defined by the diameter of the affected area [21] and scored on a five-point scale between 1 (mild) and 5 (severe), whereby 1 = 1–5 cm^2^; 2 = 5–10 cm^2^; 3 = 10–15 cm^2^; 4 = 15–20 cm^2^; and 5 > 20 cm^2^.

### 2.3. Behaviour

The behaviour of flystruck and non-flystruck sheep was analysed from footage collected in the paddock using a hand-held video camera (Panasonic HC-W570M: Panasonic Corporation, Kadoma, Japan) in a fixed position approx. 50 m from the mob. To avoid sampling bias, filming of focal animals within the mob for behavioural assessment occurred 10 s after the trained personnel visually identified suspected flystruck and no-flystruck sheep. Although flystrike may occur without faecal soiling of the breech region, to avoid visual discrimination of the flystruck status of animals by observers, where possible footage was predominately captured from the front and side of animals. After physical inspection and, if required, treatment, sheep were returned to the group and allowed to settle for 10–20 min to minimise potential effects of disruption before stock were again surveyed for filming. Footage collected on-farm was subsequently reviewed and edited to depict sheep for analysis. Resulting video clips were approximately 20 s duration (20.5 ± 0.6 s). To ensure that the results of the quantitative behavioural scoring were comparable with those from the QBA analysis, both quantitative and qualitative behavioural assessments were conducted on these video clips.

#### 2.3.1. Quantitative Behaviour Scoring

Three experienced observers blinded to the treatment groups scored footage for the 16 sheep for the incidence of abnormal behaviour (Table 1a), and the number of sheep engaged in each of these activities was calculated for each treatment group. A score of ‘restlessness’ was calculated for each animal as the total number of interruptions or changes to the predominant behaviour (Table 1b). The percentage of total time each individual animal spent walking, grazing, total standing, and standing with an abnormal posture was also recorded (Table 1c); every animal was considered to be engaged in one of these activities for the duration of observation.

#### 2.3.2. Qualitative Behavioural Assessment (QBA)

A total of 26 observers were recruited from Murdoch University staff and students (17 female, 8 male and 1 unidentified) to assess the videos using the free-choice profiling (FCP) methodology. Observers were blind to the study objectives and treatments investigated at time of assessment. All but two observers were naïve to the QBA methodology, with the two observers indicating that their previous experience with the QBA methodology was used to assess non-sheep species. Removal of these two observers did not alter the overall significance of the consensus and all observers fell within the 95% confidence interval, indicating the high level of agreement between observers in their use of descriptive terms to quantify the behavioural expression of sheep. Thus, all 26 observers were retained in the consensus. 

Observers completed a short survey regarding their past experiences with sheep and other domestic livestock species upon initial contact. Of the 26 observers, 16 (61.5%) were classified as completely inexperienced with sheep, indicating that they had never spent time working with sheep. Nine (34.6%) had limited experience working with sheep. The remaining observers (3.8%) indicated that they were experienced, having worked with sheep for more than a year.

Observers were required to attend two sessions—a term generation session followed by the quantification session. Observers were given detailed instructions on completing the QBA scoring sessions but were not given any details about the animals or the experimental treatments. 

##### Session 1—Term Generation and Training

Observers were shown 10 video clips (average 26 ± 5 s duration), which were not used in the assessment session, that depicted sheep performing a wide range of behavioural expressions, experimental and environmental conditions, to allow observers to describe as many aspects of the sheep’s expressive repertoire as possible. Briefly, observers were shown examples of both healthy (*n* = 5) and unhealthy sheep (*n* = 5), where video clips included sheep that were in isolation or in groups, within a test arena or in paddock, and/or were flystruck, lame, inappetent, had high or low faecal egg counts, or were healthy (footage was collected over a range of treatments, including those used for other QBA studies [22]). After watching each video clip, observers were given 2 min to write down terms they thought described the animal’s behavioural expression. There was no limit imposed on the number of descriptive terms an observer could generate, but terms needed to describe how the animal behaved (e.g., nervous, relaxed), rather than ‘what’ the animal was doing (i.e., physical descriptions of the animal such as vocalising, chewing, tail flicking). Subsequent editing of the descriptive terms was carried out to remove terms that described actions, and terms that were in the negative form were transformed to the positive for ease of scoring and to ensure consistency between observers (e.g., ‘unhappy’ became ‘happy’). The result was a unique list of descriptive terms for each of the 26 observers to be used for quantification in Session 2. For observers to score sheep, each unique descriptive term was attached to a visual analogue scale (minimum to maximum expression of that term) in an electronic worksheet (Microsoft Excel 2003, North Ryde, NSW, Australia) and for each observer the list of terms was randomly arranged within this worksheet.

##### Session 2—Quantification

Observers viewed and scored video clips of the 16 assessment sheep using their own unique lists of descriptive terms. Observers were instructed to score each animal’s expression using the visual analogue scale by placing an ‘x’ at the appropriate point between the two extremes of the scale bar, where minimum (= 0) reflected the absence of expression of that particular descriptive term, and maximum (= 100) indicated that the animal could not show an expression more strongly. The distance between the minimum-point and their mark on the scale reflected the intensity of each animal’s expression on that term.

### 2.4. Statistical Analysis

All statistical analyses were carried out using GenStat 18 (Genstat 2018, VSN International, Hemel Hempstead, Hertfordshire, UK) and Excel for Windows 2016 (Microsoft Inc, Redmond, WA, USA). All data were tested for normality (Shapiro–Wilk tests), and where required, non-parametric analyses were used.

#### 2.4.1. Quantitative Behaviour Scoring

Inter-observer reliability and the concordance between the three observers were evaluated by Kendall’s coefficient of concordance. Chi-square tests were used to compare count data between treatment groups (flystruck or non-flystruck animals) for the four abnormal behaviours recorded. A Students *t*-test was used to investigate differences in total restlessness scores between groups. For the behavioural time-budget categories, Mann–Whitney *U* tests were used to identify differences between treatment groups. In all cases, the standing observed in assessment animals was classified as Abnormal, consequently Total Standing was not reported herein.

#### 2.4.2. Qualitative Behavioural Assessment (QBA) Analysis

For QBA, the distance from the start of the visual analogue scale to where the observer had made a mark for each term was measured (where minimum = 0 and maximum = 100) and these data were analysed by means of Generalised Procrustes Analysis (GPA) (Genstat 2008, VSN International, Hemel Hempsteat, UK; [16]).

For a detailed description of GPA analysis and output interpretation procedures, see Wemelsfelder and colleagues [16].

Briefly, GPA is a multivariate technique that identifies underlying patterns in observer assessments (i.e., descriptive terms of the animal’s behavioural expression) and calculates the level of consensus between observer assessments of the individual animals. The statistical process whereby this best-fit pattern, termed the consensus profile, is identified takes place independently of the meaning of descriptive terms used by observers. The percentage of variation between observers (in their assessment of individual sheep) that is explained by the consensus is captured as the Procrustes statistic. The statistical performance of the consensus profile above chance is calculated by comparing (using a one-sample *t*-test) the Procrustes statistic to the mean of a simulated distribution of 100 Procrustes statistics generated through 100 iterations of the analysis, where the data is randomised in a different permutation each time. Significance values in that test of *p* < 0.001 or better can be taken as evidence that the consensus profile was not a methodological artefact and represents a common pattern identified by observers. As an additional measure of inter-observer reliability between observers, scores for each individual clip were correlated using Kendall’s coefficient of concordance *W*.

The consensus profile is then simplified to a smaller number of dimensions (in this case, two), explaining the majority of variation between observed animals, by Principal Component Analysis (PCA). To allow semantic interpretation of these main dimensions, the individual observer’s terms with the strongest correlation coefficients with the consensus dimension scores were identified. This process was entirely post hoc to the computation of the consensus profile.

Students *t*-tests were used to test for a treatment effect (flystruck or non-flystruck animals) on the average scores for each of the sheep on these two GPA dimensions. Demographic information was presented to demonstrate the level of experience QBA observers had with sheep but was not statistically analysed.

#### 2.4.3. Associations between Breech Soiling Assessments, Behaviour Scoring, and QBA

A PCA based on standardised variables was used to investigate the relationship between the behavioural, both quantitative and qualitative, and breech soiling data. Variables were interpreted according to their loadings on the most important components (PCA 1 and 2), and variables with high loadings on the same component can be grouped. Such groupings indicate which of the variables recorded were most closely related and those that had the greatest association with the treatment groups. This served to determine whether the GPA dimensions were associated with known behavioural parameters of stress and physical indicators of flystrike and poor welfare. The PCA (correlation matrix, no rotation) was performed by analysing individual animal data on all 14 variables (8 behavioural parameters, 4 breech soiling measures and individual animal scores on QBA dimensions 1 and 2). A urine stain score was missing for one animal from the flystruck group, thus the corresponding animal was not submitted to the PCA. Spearman rank order correlations were also employed to examine the association between the collected parameters.

## 3. Results

### 3.1. Breech Soiling Assessment

Average dag scores of flystruck animals at time of treatment were 3.25 ± 0.37 (mean ± s.e.m.; range: 2–5), indicating moderate to high episodes of diarrhoea. Average dag moisture content was moderate with sheep recording scores of 2.00 ± 0.20 (mean ± s.e.m.; range: 1–3). Urine stain scores were moderate across the assessed animals, averaging 2.14 ± 0.24 (mean ± s.e.m.; range: 1–3). Severity of flystrike was not very high, with five animals (62.5%) having a severity score of 2 (size of flystrike: 5–10 cm^2^) and the remaining three animals (37.5%) receiving moderate scores of 3 (size of flystrike: 10–15 cm^2^). Although none of the recorded flystrikes were classified as mild (being less than 5 cm^2^), there were no scores indicative of severe flystrike.

### 3.2. Quantitatve Behaviour Scoring

Table 2 shows the Kendall’s coefficient of concordance (*W*) for the degree of agreement between the three observers who analysed the behaviour of sheep from video footage. These results show that for each behaviour recorded, the observers showed significant agreement on the ranking of the focal sheep. Given this result, in subsequent analysis of quantitative behaviour, we considered only the data of one trained observer scoring the 16 sheep observed.

There was a significant treatment effect in the number of animals exhibiting abnormal behaviour (Table 3a). More of the flystruck sheep turned their head (*p* = 0.005) and actively bit their rump region (*p* = 0.002) compared to the non-flystruck animals (none performed either of these behaviours). In addition, although kicking and tail wagging was also observed in the non-struck animals, more of the flystruck sheep were observed to display these behaviours compared to the non-flystruck animals (*p* = 0.015 and *p* = 0.011, respectively).

Overall restlessness also showed a significant treatment effect, with flystruck animals recording a 3.9-fold increase in interruptions to normal grazing behaviour (including kicking, tail wagging, head shaking, head turning and biting rump) compared to non-flystruck animals (F_14_ = −4.77, *p* < 0.001; Table 3b).

There were also significant treatment effects for the percentage of time sheep spent walking, grazing and standing, with flystruck sheep spending less time grazing (*U* = 4, *p* = 0.002) and more time standing with abnormal posture (*U* = 3, *p* = 0.001) than non-flystruck animals (Table 3c). There was no significant treatment effect for the proportion of time the sheep spent walking (Table 3c).

### 3.3. Qualitative Behavioural Assessment (QBA)

The 26 observers generated a total of 66 unique terms to describe the sheep they were shown (average 13.5 ± 3.4 terms per observer; range 8–21) using the FCP methodology. The GPA consensus profile explained 51.4% of the variation between observer scores of sheep and this differed significantly from the mean randomised profile (*t*_99_ = 34.89, *p* < 0.001). Two main dimensions of behavioural expression were identified, explaining a total of 67.7% of the overall variation in scores attributed to individual sheep (Table 4). Observers showed a moderate to good level of agreement for the two main dimensions, with Kendall’s W values of 0.56 and 0.66, respectively (Table 4).

The word charts for the observers appeared to be semantically consistent across the observers. Terms with the strongest positive and negative loadings within each of the GPA dimensions are shown in Table 4. Low values for GPA dimension 1 were associated with descriptive terms such as ‘exhausted’ and ‘irritated’, and high values with terms such as ‘positively occupied’ and ‘assured’. For GPA dimension 2, low values were associated with descriptive terms such as ‘indecisive’ and ‘depressed’, and high values with terms such as ‘inquisitive’ and ‘collected’.

**Figure 1:**
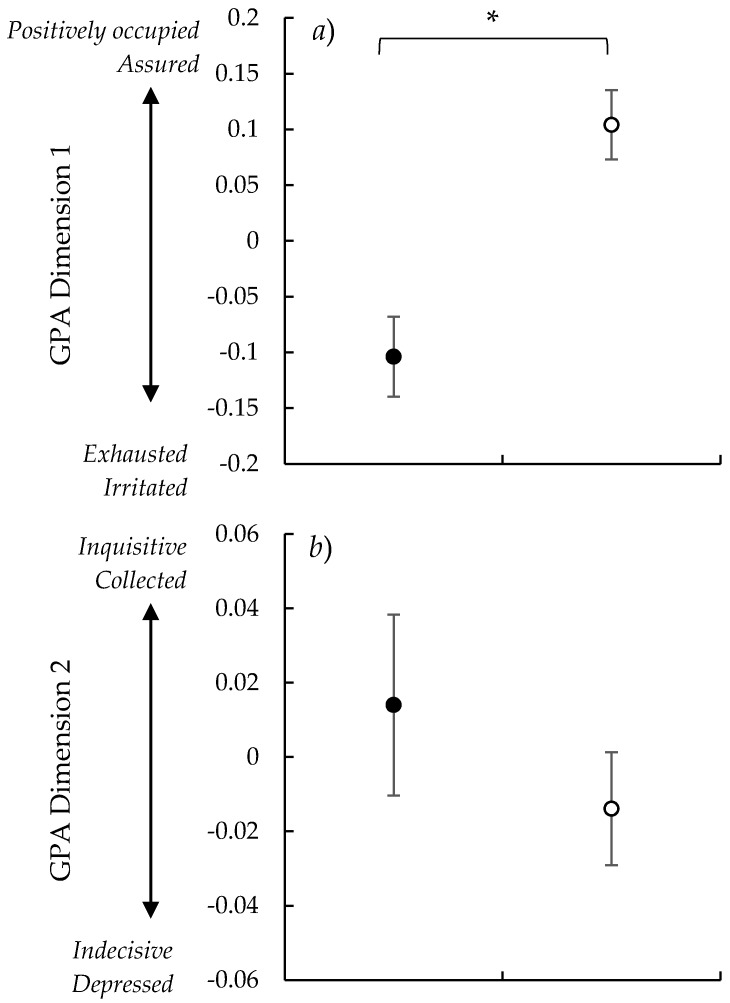
Effects of the treatment groups; flystruck (*n* = 8) and non-flystruck (*n* = 8) on General Procrustes Analysis (GPA) scores for Qualitative Behavioural Assessment (QBA) (*a*) Dimension 1; and (*b*) dimension 2 assessed from video footage taken of sheep in paddock. Values are means ± S.E.M. Asterisks (*) indicate significant difference between treatment groups (*p* < 0.05).

Flystruck sheep were scored as significantly more ‘exhausted/irritated’ on GPA dimension 1 compared with non-flystruck sheep, which were scored as more ‘positively occupied/assured’ (Figure 1a) (*t*_14_ = 4.38, *p* < 0.001). There were no significant differences between the two treatment groups on GPA dimension 2 (*t*_14_ = −0.97, *p* = 0.35).

The three highest weighting terms for each GPA dimension were selected for the purpose of labelling the GPA dimensions and describing the dimensions in relation to the experimental group (Figure 1).

### 3.4. Association between QBA, Behavioural and Breech Soiling Parameters

**Figure 2:**
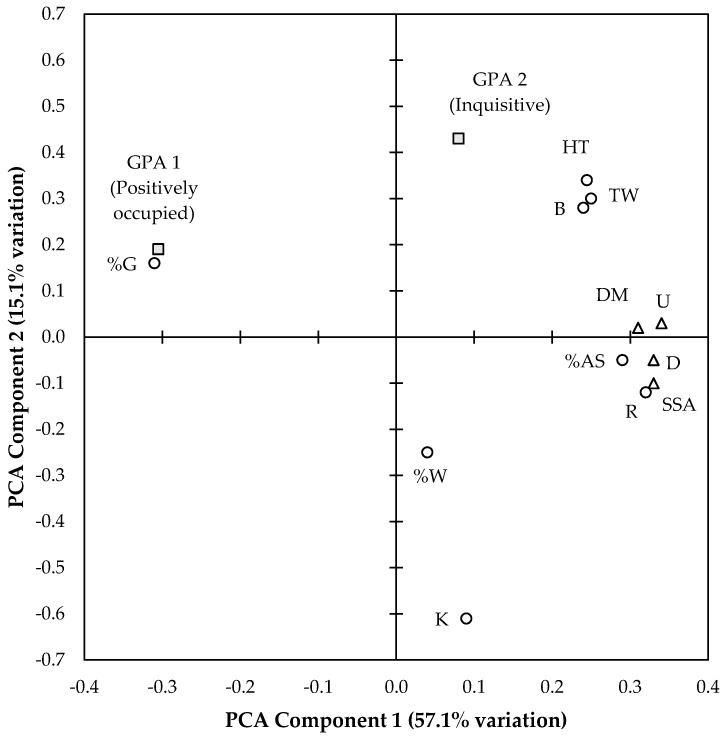
Summary of Principal Component Analysis (PCA) dimensions comparing scores for breech soiling variables, quantitative behaviour scoring, and Qualitative Behavioural Assessment (QBA). Breech soiling variables (triangles): Dag score (D), Dag moisture score (DM), Urine stain score (US), and Size of flystruck area (SSA). Quantitative behaviour scoring (circles): Abnormal standing (AS), Grazing (G), Biting rump region (B), Head turn (HT), Kicking (K), Tail wagging (TW), Restlessness (R), and Walking (W). QBA (squares): Generalised Procrustes Analysis (GPA) scores on dimensions 1 and 2.

QBA scores were analysed together with breech soiling measures and quantitative behaviour measures through PCA, revealing two main components explaining 50.2% and 14.6% of the variation between sheep, respectively. Figure 2 shows the loadings of the 14 variables on these two main components. On PCA component 1, the first GPA dimension (‘positively occupied’; −0.31) and percentage grazing (−0.31), showed the highest negative loadings, whereas urine stain scores (0.34), dag scores (0.33), size of flystruck area (0.33), restlessness (0.32), dag moisture scores (0.31), abnormal (0.29), head turning (0.24), tail wagging (0.25) and biting rump region (0.24) showed the highest positive loadings. These associations are supported by significant correlations between individual QBA dimension 1 scores, indicating that sheep that spent less time grazing (R_s_ = 0.79; *p* < 0.001), more time standing abnormally (R_s_ = −0.76; *p* < 0.001), displayed higher levels of overall restlessness (R_s_ = −0.75; *p* < 0.001), and engaged in kicking (R_s_ = −0.35; *p* < 0.05), head turning (R_s_ = −0.56; *p* < 0.01), and biting rump region (R_s_ = −0.61; *p* < 0.01) were perceived by observers as more ‘exhausted/irritated’. In addition, size of flystruck area (R_s_ = −0.79; *p* < 0.001) and dag score (R_s_ = −0.51; *p* < 0.01) were negatively correlated with GPA dimension 1 scores. On PCA component 2, kicking (−0.61) and percentage walking (−0.25) showed the highest negative loadings, whereas GPA 2 scores (‘inquisitive’; 0.43), head turning (0.34), tail wagging (0.30), biting rump region (0.28) and GPA 1 scores (‘positively occupied’; 0.19) showed the highest positive loadings. These associations were partially supported by significant correlations between individual QBA dimension 2 scores, where sheep that spent more time walking (R_s_ = 0.43; *p* < 0.05), and tended to engage in tail wagging (R_s_ = 0.50; *p* < 0.05), head turning (R_s_ = 0.38; *p* < 0.05), biting rump region (R_s_ = 0.39; *p* < 0.05), and kicking (R_s_ = −0.34; *p* < 0.05), were perceived by observers as more ‘inquisitive/collected’.

**Figure 3:**
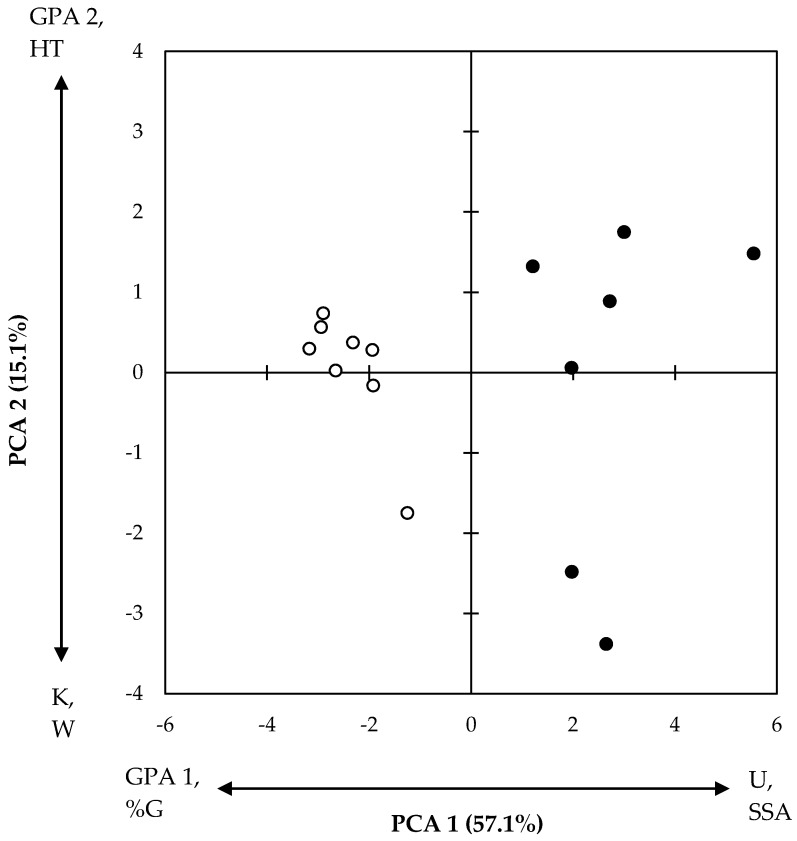
Position of individual sheep from the flystruck (closed) and non-flystruck (open) groups on the two main principal component analysis (PCA) dimensions characterised by Urine stain score (U), Size of flystruck area (SSA), GPA 1 (Positively occupied), Grazing (%G), Kicking (K), Walking (%W), GPA 2 (Inquisitive) and Head turning (HT). Complete loadings for PCA dimensions 1 and 2 are summarised in Figure 2.

As done during the GPA process, the positions of the assessment animals were plotted to summarise the assessment of these animals on the basis of all recorded variables (Figure 3). The distribution of these sheep on the two main PCA components was interpreted using the PCA configuration and loadings presented in Figure 2, such that the components were characterised by those variables with the highest and lowest loadings. This plot shows a clear grouping of sheep by treatment on PCA component 1 (Figure 3).

## 4. Discussion

The diagnosis of flystrike is usually straightforward based on physical examination of individual sheep, but there is an obvious need for a non-intrusive method of identification that does not involve the gathering and individual handling of all animals. Such a method would allow for the targeted or selective treatment of animals, reducing costs and minimising labour as it would involve only the gathering, handling and treatment of flystruck animals rather than all stock. Here, we applied both quantitative and qualitative methods to study the behaviour of breech-flystruck sheep in a paddock setting to identify potential indicators of flystrike. The significant finding of this study was that flystruck sheep displayed observably different behaviour compared with non-flystruck sheep. First, flystruck sheep displayed different behaviour profiles, characterised by more restless behaviour, the adoption of abnormal postures and reduced grazing, indicating that flystrike causes distress. Second, flystruck animals displayed an ‘exhausted/irritated’ demeanour as assessed by observers who were blinded to the experimental treatment. Third, the behaviour scoring and demeanour of animals were associated with physical flystrike parameters (breech soiling variable; size of flystruck region and dag score), highlighting the biological relevance of these observations. Together, these findings suggest that non-intrusive monitoring of specific behaviours in a formal manner may prove a useful tool for producers to identify breech-flystruck sheep.

### 4.1. General Quantitative Behavioural Responses of Sheep to Breech Flystrike

Only flystruck sheep bit or attempted to bite their rump region in the present study, suggesting that such behaviour is of pathological origin. The prolonged or intense attention to an area in the form of biting, licking or rubbing can be an indicator of pain and distress in animals [23]. Such behaviour may be an attempt to alleviate painful, itching and/or irritating sensations, or perhaps represents an attempt by the sheep to remove the source of the noxious stimulus caused by feeding activity of the maggots, accumulative damage to the skin and underlying tissues, and ensuing inflammatory response. These biting behaviours were distinctive and only occurred in flystruck sheep, suggesting that they could prove to be useful indicators of breech flystrike.

Flystruck animals also displayed more ‘restless’ behaviour than non-flystruck sheep, having a near 4-fold increase in the expression of abnormal behaviours that interrupted their predominant behaviour (walking/grazing/standing). The combination of kicking, head shake, head turn, biting rump region, and tail wagging provided a good index for the identification of sheep with flystrike in the present study. These results are consistent with studies of behavioural responses of lambs to painful husbandry procedures [24,25,26,27], where combined or integrated scores were useful in distinguishing pain responses in lambs. It is thought that the cumulative scoring of active behaviours into a single index, such as the present restlessness score herein, may compensate for differences in the expression of the pain response between individuals subject to the same challenge [25]. Moreover, such scores may also compensate, to some extent, the fact that animals can display such behaviours as part of their normal repertoire. This was seen in the present study with both flystruck and non-flystruck animals observed to kick, wag their tails and shake their heads, although fewer non-struck animals tended to display such behaviour. Perhaps a combined behaviour index that reflects the intensity of expression would prove more useful for the identification of flystrike than simply quantifying separate behaviours. This may also allow for easier discrimination between animals and compensate, at least in part, for the occurrence of behaviours in unchallenged animals.

Flystruck sheep spent less time grazing than non-flystruck sheep, and tended to adopt abnormal postures while standing. Reduction in grazing time was a consequence of the repeated expression of abnormal, presumably pain-related, behaviours, as evidenced by extreme ‘restlessness’ observed in the flystruck animals. Broadmeadow, Connell and O’Sullivan [8] report that flystrike is accompanied by high fever, resulting in reduced feed intake in sheep. Fever and inappetence could contribute to the observation of reduced grazing in our study. From a welfare perspective, reduction in grazing, a behaviour that animals would be considered highly motivated to perform, is both important and concerning. Flystruck sheep also spent a larger proportion of time standing abnormally, which is consistent with the adoption of abnormal postures when standing in lambs in response to castration and mulesing [25,27,28], indicating discomfort and/or pain. While observations of grazing and abnormal postures may provide useful information in terms of welfare, they are neither unique to breech flystrike occurring in a broad range of situations, nor feasible due to the amount of time such observation would require. Thus, for such measures to prove useful in the identification of flystruck animals for treatment they would need to be incorporated into a larger assessment scheme and be remotely captured.

Not all of the behavioural parameters recorded in the present study proved useful. Indeed, several of the proposed pain-related behaviours (kicking/head turn) were observed infrequently in the flystruck group (less than 50% of animals) or were also observed in the unchallenged, non-flystruck group. It is important to consider that most of the behavioural indicators of pain in sheep were developed to assess lambs undergoing common painful husbandry procedures (e.g., castration, tail docking and mulesing). As such, they may not adequately reflect pain responses in adult animals or the type of injury and presumed pain caused by the disease.

### 4.2. Associations between Breech Soiling Assessments, Behavioural Scoring and QBA

Quantitative behavioural scores and scores for behavioural expression (QBA) converged towards the same interpretation, indicating that flystruck sheep are more distressed compared with non-flystruck animals. The behavioural response of sheep suffering breech flystrike is characterised by an ‘exhausted/irritated’ demeanour, comprising the expression of a combination of different active behaviours with abrupt movement of the head, tail or limbs (head turning, biting, tail wagging and restlessness), the adoption of abnormal postures while standing and reductions in grazing behaviours. Furthermore, analysis indicates that these responses are related to the irritation or pain caused by tissue damage (strike severity scores) and degree of breech soiling in the animal (dag score and urine score), highlighting the biological relevance of these assessments. How individual sheep expressed pain or irritation caused by flystrike in the present study appeared to be variable; consequently, the complex behavioural profile of animals suffering breech flystrike may have been difficult of capture or interpret using purely quantitative methods. Such variability was also seen in the pain responses of lambs to rubber ring castration and tail docking [25]. That observers were able to successfully differentiate animals using QBA, and that those scores combined meaningfully with quantitative data in the present study, adds value to QBA methodology as a holistic, ‘whole-animal’ approach. Thus, QBA descriptors are suited to pick up responses to breech flystrike that may go undetected when behaviour is quantified in an isolated manner (e.g., quantification of kicking or head turning events). It appears that a combined score of those behaviours indicative of restlessness and assessment of demeanour using QBA provided the best individual discriminatory basis between animals and may be reliable indicators for the practical identification of breech flystrike in sheep.

### 4.3. Qualitative Behaivoural Assessment (QBA)

Differences in behavioural expression were observed between two groups of sheep, where observers scored the flystruck sheep as more ‘exhausted/irritated’ compared to non-flystruck animals (which were scored as more ‘positively occupied/assured’). Changes caused by immunological responses designed to counter infestation and associated toxaemic challenge, fever in particular, are often energetically costly [29,30], and affected animals may consequently appear lethargic or fatigued and uninterested in their surroundings. Sick animals are frequently described as lethargic or depressed and this lethargy is generally associated with the fever response [31]. It is likely that the ‘irritated’ demeanour in flystruck animals reflects the negative emotional state associated with the presence of painful cutaneous lesions or the active feeding of the maggots causing irritation, pain or discomfort. Disease and injury are generally considered to have emotional components and studies have suggested that QBA scores can reflect the deleterious effect that injury or disease has on emotional state in pigs [32], cattle [33] and sheep [34]. While we cannot directly observe or assess psychological welfare, it has been suggested that QBA offers insight into emotional (psychological) state by summarising how animals perceive and interact with their environment through assessments of body language or behavioural expression [35,36]. The relationship between emotional state, behavioural expression and disease may not be a simple one, but it is clear from these results that breech flystrike had a negative effect on the animals that led to altered behavioural expression as identified by observers using the QBA methodology.

### 4.4. Considerations for Practical Application

From a practical viewpoint, the development of a small number of indices that indicate flystrike remotely would be ideal for sheep producers. Although preliminary in nature, the results herein suggest that while the behavioural presentation of flystrike varied between animals, the common denominator amongst the affected animals was the presence of agitation or restlessness, as evidenced by the intense expression of restless behaviours and the ‘exhausted/irritated’ demeanour. It appears that the altered behavioural expression associated with breech flystrike is obvious and easily identifiable from video footage as short as 20 s duration. Indeed, whether in person or from remote video footage, observations required for QBA are considered quicker than traditional behavioural recording [22,34,37,38,39]. Moreover, QBA has the potential to act as an initial screening tool [18], thus when used during routine monitoring may provide producers an early warning of flystrike and would allow producers to identify those animals in need of closer evaluation and, if required, treatment. Overall, it is likely that stockpersons informally use these behaviours to inform choices to visually inspect stock for flystrike, and the work presented herein validates these observations. Perhaps the incorporation of formal scoring and training to improve recognition of these behaviours would advance observational skills and facilitate the more prompt and accurate identification of animals in need of treatment on-farm.

The use of the comprehensive assessment of body language provided by QBA coupled with the more specific information offered from behavioural indices of restlessness and grazing is well suited to automated capture. There have been remarkable advancements and success in the development of biosensors to detect behavioural changes across various species [40,41,42]. It is possible that such technologies could be adapted to detect those behaviours presented here. For example, Williams, et al. [43] reported preliminarily data that suggest accelerometers attached by way of neck collars could identify drinking behaviour in cattle though the detection of head–neck position changes. Perhaps this or a similar technology could be modified to allow for the identification of postural change of the neck, indicative of orientation of head biting the rump region, and/or to quantify grazing behaviour.

### 4.5. Limitations and Future Work

Several additional steps need to be taken in order to assess the potential relationship between behavioural expression and breech-strike, and to further conclude on the diagnostic value of behavioural indices for practical identification of flystruck sheep for treatment. For example, as the quantitative behavioural observation in the present study was based on short observational periods, a more detailed observation of the behaviour in a larger sample of flystruck animals over a longer period of time is needed to classify behaviours as definitively indicative of breech-flystrike. Furthermore, a study investigating changes in the behaviour, behavioural expression and production performance of sheep suffering strike of varying severity (degrees of damage to tissues) would be valuable to identify behaviours for early detection. Likewise, the inclusion of physiological parameters is vital for the understanding and support of the findings in the present study. For example, assessments of the activity of the sympathetic nervous system such as heart rate and respiration, in addition to traditional measures of the hypothalamic–pituitary–adrenal system could be valuable as they are considered indicators of pain and distress, and have been applied across a variety of animal models (reviewed by [44,45]). It is also crucial to consider that there are different types of flystrike that need to be monitored on-farm and these types of flystrike may impact the behaviour of sheep or present differently. Since accurate monitoring of stock for all types of flystrike is necessary on-farm, this is an area where future work is required. Nonetheless, the data presented here provide proof of concept that assessments of behaviour provide useful information that could be used to identify breech-struck sheep on-farm.

## 5. Conclusions

In the present study, flystruck animals spent less time grazing, had abnormal posture when standing, exhibited head turning and biting of the rump region, displayed higher levels of restlessness, and were perceived to be more ‘exhausted’ and ‘irritated’ compared to non-flystruck sheep. These behaviours are likely to be pain-related and these results, in agreement with general considerations, suggest that flystrike is painful and causes clear distress in sheep. More extensive studies are still required to further validate the relationship between flystrike and these pain-related behaviours, and to refine the use of these behaviours to create an index for the identification of flystrike. However, the results of this study suggest that the behaviour of breech-flystruck sheep diverges markedly from normal patterns and these differences are easily observable. Thus, the formal monitoring of animals for these behaviours represents a potential tool for the initial remote identification of sheep suffering breech-strike.

## Figures and Tables

**Table 1:** Description of behaviour used to score sheep from 20 s video clips; (a) abnormal behaviour, (b) restlessness, and (c) percentage of time spent waling, grazing and standing.

Behaviour	Description
(a) Abnormal behaviour (count of sheep per treatment group showing each of these activities at least once over the clip duration)	
Kicking	Either front or hind limb was raised and forcefully strikes the ground or is moved backwards or forwards without moving other limbs.
Tail wagging	Rapid and repetitive side-to-side tail movements separated from another tail wagging event by at least 2 sec.
Head turning	Turning head beyond the shoulder.
Biting rump region	Turing head beyond the shoulder and sheep actively biting rump area.
(b) Score of ‘restlessness’	
Restlessness	The number of interruptions or changes to the predominant behaviour (walking, grazing or standing) of the sheep for the combined (abnormal) behaviours of kicking, head shake, head turning, biting rump region and tail wagging.
(c) Percentage of time spent (% of total time observed)	
Walking	Moving forward in a four beat motion for 2 s or more with head orientated in direction of movement.
Grazing	Actively chewing pasture. Head may be lowered towards ground or raised if chewing.
Total standing	Standing stationary on four legs, without jaw movement indicative of chewing. Includes normal standing with head and neck in normal or neutral position, and abnormal standing (see below) restricted to when animal was not actively grazing.
Abnormal standing	Abnormal head and neck posture when standing stationary includes standing with head lowered (below withers) and hunched back, head orientated towards the side, neck extended and head in low position or head turned towards rump region. Restricted to when animal was not actively grazing.

**Table 2:** The degree of agreement between observers on video-based assessment of sheep behaviour.

Behavioural Parameters	Kendall *W*	*p*-Value
(a) Abnormal behaviour		
Kicking	0.77	0.003
Tail wagging	0.84	<0.001
Head turn	0.82	0.001
Biting rump region	1.00	<0.001
(b) Score of ‘restlessness’		
Restlessness	0.89	<0.001
(c) Percentage of time spent		
Walking	0.94	<0.001
Grazing	0.90	<0.001
Abnormal standing	0.84	<0.001

**Table 3:** Comparison of behavioural scoring for sheep in flystruck and non-flystruck treatment groups taken from video clips of 20 s duration.

Behavioural Parameters	Raw Value	
	Flystruck (*n* = 8)	Non-flystruck (*n* = 8)
(a) Abnormal behaviour (count of sheep per treatment group)		
Kicking	3 (37.5%) a	1 (12.5%) b
Tail wagging	5 (62.5%) a	1 (12.5%) b
Head turn	4 (50.0%) a	0 (0.0%) b
Biting rump region	5 (62.5%) a	0 (0.0%) b
(b) Score of ‘restlessness’ (no. instances)		
Restlessness	4.89 ± 0.55 a	1.25 0.53 b
(c) Percentage of time spent (% of total time observed)		
Walking	0.17 ± 0.08 a	0.15 ± 0.06 a
Grazing	0.09 ± 0.09 a	0.74 ± 0.07 b
Abnormal standing	0.73 ± 0.11 a	0.12 ± 0.07 b

Values are percentages of sheep per treatment exhibiting each behaviour or mean ± s.e.m. For abnormal behaviours, different letters indicate significant differences between treatment groups using Chi-Squared (at *p* < 0.05), whereas different letters indicate significant differences using Students *t*-test and Mann–Whitney *U*-tests (at *p* < 0.05) for restlessness and percentage of time spent walking, grazing and standing abnormally, respectively.

**Table 4:** Terms used by observers using the Free-choice Profiling (FCP) method of Qualitative Behavioural Assessment (QBA) to describe the behavioural expression of sheep filmed in paddock.

GPA Dimension (% of Variation Explained) Kendall’s *W*	Descriptive Terms †	
	Low Values	High Values
GPA 1 (58.1%) 0.66 ^***^	Exhausted (1), irritated (8), tense (1), agitated (7), frustrated (4), distressed (8), alarmed (1).	Positively occupied (1), assured (1), unhurried (1), active (1), content (12), relaxed (21), settled (2), happy (18).
GPA 2 (9.6%) 0.56 ^***^	Indecisive (1), depressed (2), afraid (1), insecure (2).	Inquisitive (4), collected (1), dull 1), interested (2), lively (2), aggressive (3), alarmed (1), unhurried (1), joyful (2), settled (2), cautious (8).

^***^p < 0.001. † Terms that had strong loadings with the Generalised Procrustes Analysis (GPA) dimensions are listed. Terms shown have loadings of > 0.6 (high values) and < −0.6 (low values) for GPA dimension 1, and >0.3 (high values) and < −0.3 (low values) for GPA dimension 2. Numbers in parentheses represent the number of observers that generated and subsequently used that word to assess sheep expressive behaviour.
